# Evaluation of Suicide Risk Assessment Documentation Standards in Psychiatric Outpatient Clinics

**DOI:** 10.1192/bjo.2026.11702

**Published:** 2026-06-30

**Authors:** Ebtesam Honarvar, Farhana Ashraf, Andrea Pathak, Ashish Pathak

**Affiliations:** Essex Partnership University NHS Foundation Trust, Basildon, United Kingdom

## Abstract

**Aims:**

To evaluate the quality of suicide risk assessment documentation in outpatient consultations against NICE NG225 and Local Trust standards, and to assess whether a focused feedback intervention improved documentation quality.

**Methods:**

A retrospective baseline audit reviewed the first 50 consecutive outpatient consultations from 1 December 2025. Findings were summarised and fed back to clinicians. A re-audit examined 50 subsequent consultations from 23 January using the same structured assessment criteria. Core criteria included documentation of suicidal ideation, plan, intent,access to means, history of self-harm, risk formulation, and safety planning. Additional criteria assessed shared decision-making and communication with the General Practitioner.

**Results:**

Substantial improvement followed the feedback intervention. Documentation of consent and confidentiality increased markedly (from 4% to 76%), reflecting greater attention to patient involvement and transparency. Recording of history of self-harm, a key predictor of future risk, improved significantly (from 28% to 76%). Protective factors, often under-recorded locally, increased from 52% to 78%, strengthening overall risk formulation. General risk formulation itself showed a notable improvement (62% to 82%), showing a clearer integration of dynamic and historical factors.

Among patients expressing suicidal ideation (baseline 28%, re-audit 50%), documentation of suicidal plan and intent improved from 0% at baseline to 84% post-intervention–representing one of the most clinically meaningful changes observed. Despite improvement in several domains, access-to-means exploration remained low (6% to 14%), identifying an important area requiring further attention. Documentation of shared decision-making showed some decline (98% to 84%), suggesting that emphasis on risk-specific domains may have inadvertently reduced focus on collaborative discussions.

**Conclusion:**

Targeted feedback led to clear and meaningful improvements in several domains central to high quality suicide risk assessment, particularly self-harm history, protective factors, overall risk formulation, and documentation of plan and intent among patients with suicidal ideation. Persistent gaps in assessing access to means highlight a priority area for additional intervention. Future work would focus on embedding structured reminders within clinical templates, reinforcing collaborative practice, and conducting further re-audits to sustain improvement.

